# PTCOG Ocular Survey – Perspective on Ocular Particle Therapy: Current Practices and Emerging Trends

**DOI:** 10.1016/j.ijpt.2026.101300

**Published:** 2026-01-29

**Authors:** Linda Mortimer, Roelf Slopsema, Alejandro Mazal, Jan-Willem Beenakker, Rémi Dendale, Andrea Denker, Emmanuelle Fleury, Anais Groulier, Andrzej Kacperek, Kavita K. Mishra, Jatinder Saini, Alexei V. Trofimov, Juliette Thariat, Petra Trnková, Marie Vidal, Helen A. Shih, Jan Hrbacek, Jens Heufelder

**Affiliations:** 1 Medical Physics Department, The Clatterbridge Cancer Centre NHS Foundation Trust, Liverpool, UK; 2 Department of Radiation Oncology, Emory University School of Medicine, Atlanta, GA, USA; 3 Centro de Protonterapia Quironsalud, Madrid, Spain; 4 Leiden University Medical Center, Departments of Ophthalmology, Radiology and Radiation Oncology, Leiden, the Netherlands; 5 HollandPTC, Delft, the Netherlands; 6 Institut Curie Proton Therapy Center, Building 101, Orsay University Campus, Orsay, France; 7 Helmholtz-Zentrum Berlin für Materialien und Energie, Protonentherapie, Berlin, Germany; 8 Erasmus Medical Center Cancer Institute, University Medical Center, Department of Radiotherapy, Rotterdam, the Netherlands; 9 Centre Antoine Lacassagne, Nice, France; 10 Department of Medical Physics and Biomedical Engineering, University College London, UK; 11 Department of Radiation Oncology, University of California, San Francisco, CA, USA; 12 Department of Radiation Oncology, University of Washington School of Medicine, Seattle, WA, USA; 13 Department of Radiation Oncology, Massachusetts General Hospital/Mass General Brigham, Boston, MA, USA; 14 Université de Caen Normandie, ENSICAEN, CNRS/IN2P3, LPC Caen UMR6534, Caen F-14000, France; 15 Faculty of Nuclear Sciences and Physical Engineering, Czech Technical University in Prague, Prague, Czech Republic; 16 Harvard Medical School, Boston, MA USA; 17 Paul Scherrer Institut, PSI, Forschungsstrasse, Villigen, Switzerland; 18 Charité - Universitätsmedizin Berlin, Department of Ophthalmology and Berlin Protonen at HZB, Berlin, Germany; 19 Centre François Baclesse, Caen, France

**Keywords:** Proton therapy, Ocular particle therapy, Uveal melanoma, Survey

## Abstract

**Purpose:**

To overview the status and trends in the current rapidly evolving field of ocular particle therapy (OPT).

**Materials and Methods:**

The Particle Therapy Co-Operative Group Ocular Subcommittee developed an online survey, including 127 questions, made available to treatment centers worldwide, between April 2022 and January 2024. The survey covered a broad range of topics from general program organization and beamline description, to diagnoses and patient numbers treated, treatment planning and set-up, quality assurance techniques, as well as insights into anticipated future developments.

**Results:**

Twenty facilities offered OPT by the end of 2023: 3 in Asia, 9 in Europe, and 8 in the USA. Combined, they treated, on average, 1800 patients per year, with uveal melanoma being the most common indication. The vast majority of treatments continue to be delivered on dedicated horizontal beamlines, both high (>120 Mev) and low (<75 MeV) energy. Five of the 7 recently established programs (n = 7, since 2015) use high-energy general-purpose beamlines, where some beam characteristics, including penumbrae size and dose delivery rate, differ from those typically achieved on dedicated beamlines. The evolution of the ocular treatment framework is ongoing as 9 centers reported the intent to upgrade their systems within five years, to engage state-of-the-art developments in beam delivery, imaging, and planning tools.

**Conclusion:**

Common protocols in dosimetry, dose, and fractionation have largely been adopted worldwide, while variability persists in planning, quality assurance, and delivery workflows. The obsolescence of dedicated ocular proton systems and key planning tools, and a lack of standardization, remain as pressing challenges. The adoption of non-dedicated high-energy systems on gantries and integration into multipurpose treatment environments enhances OPT accessibility, but this transition requires ongoing critical evaluation of dosimetric trade-offs and clinical outcomes. Multicenter collaboration, technological innovation, and clinical validation are essential to continued assurance of the safe and effective delivery of OPT worldwide.

## Highlights

• International survey results on ocular particle therapy (OPT) are presented.
• Variability in planning and quality assurance highlights a need for standardization.
• Adaptation of high-energy systems broadens access but requires clinical follow-up.
• The obsolescence of beam generation and planning systems remain pressing challenge.
• Collaboration and technical advances are essential to OPT sustainability and growth.

## Introduction

Particle therapy (PT) has a long and successful history in treating ocular lesions. Since the first proton treatment of ocular melanoma by the Massachusetts General Hospital in 1975^1^ at the Harvard Cyclotron Laboratory, nearly 50,000 eye patients worldwide at a handful of centers have been treated with protons and light ions^2^ using this highly specialized treatment technique.

In 2015, the Ocular Subcommittee of the Particle Therapy Co-Operative Group (PTCOG) conducted the first survey among PT centers with an active ocular treatment program, designed to identify the practice patterns, variability of the patient population, methods for simulation, treatment-planning, immobilization, treatment techniques, and quality assurance (QA), and the design of the beam-delivery system.^3^ Since 2015, several developments, such as doubling of centers using high-energy proton beam lines, have taken place that warrant re-evaluation of practice patterns. At the time of the first survey, all centers had a dedicated eyeline, a fixed beamline optimized to deliver small treatment fields with sharp dose gradients and at high dose rate, to seated patients. The eyeline at each center had a unique construction: 9 of the 10 centers had developed their system in-house, while only one had a commercial system. Since the previous survey, several commercial eyeline systems have become operational. Moreover, some PT centers implemented an ocular treatment program on general-purpose fixed beam lines or gantry systems alongside other treatments.^4^, ^5^ Some of the methods and techniques were adopted from the dedicated-eyeline programs, but new ones needed to be developed to accommodate the transfer to multipurpose rooms. Many approaches from non-ocular proton therapy have been applied to ocular work, for example, patients are treated supine on gantry-based systems at some institutions, computed tomography (CT) is in use for treatment planning, and magnetic resonance imaging (MRI) for target delineation^6^, ^7^, ^8^, ^9^ and 3D Monte Carlo has been introduced for dose calculation. All this has led to more divergence in practice of ocular PT. At the same time, the practice at many of the pioneer centers, where most proton eye treatments are still performed, has evolved since the original survey.^10^

To capture the status of the rapidly changing field of ocular particle therapy (OPT), the PTCOG ocular subcommittee conducted a follow-up survey. This paper summarizes and interprets these results. The report aims to share best practices between established and aspiring programs and to provide a snapshot of the current state of ocular treatments within the fast-developing landscape of PT.

## Material and methods

The survey consisted of 127 questions and was implemented as a web-based questionnaire in Survey Monkey™. Question types included multiple choice, ranking, and free text. Approximately 80% of questions were the same as those used in the 2015 survey to identify practice changes; the remaining were new to better understand current practices and capture new developments.

Among over 100 PT centers in operation worldwide,^11^ 26 were identified as having an active ocular program, actively developing, or planning to develop one within the next 5 years and were invited to complete the survey.

The survey comprised 2 main parts: Part 1: Current Practices, completed by operational centers, consisted of 9 subsections (Respondent Identifiers, Patient Mix and Prescription, Program Organization, Patient Set-Up and Immobilization, Patient Simulation and Tumor Visualization, Treatment Planning, Patient Alignment and On-line Monitoring, Proton (Ion) Therapy System, Dosimetry and QA); Part 2: Future Outlook, completed by all centers.

## Results

Twenty-three facilities responded to the survey between April 2022 and January 2024. Twenty of these were operational by the end of 2023.

### Operational centers and treatment statistics

Centers with an operational OPT line included 3 from Asia, 8 from North America, and 9 from Europe. Two centers reported using carbon ions, and one reported using helium ions for some ophthalmic treatments. The starting year of OPT for the 20 centers ranges from 1984 to 2023 (Table 1). Seven centers started their treatment activity after the last survey was conducted in 2015.^3^ Three centers were not yet operational.

**Table 1:** Overview of 20 OPT centers, their technical layout, beam parameters, and patient numbers (average number of ocular patients treated per year from 2015 through 2023, inclusive, and total treated from year of opening to end 2023).

Number	Institution	City (Country)	Accelerator	Beamline (nozzle type)	Beam delivery	Treatment position	Manufacturer of ocular beam line	Distal fall off 90% to 10% (mm)	Lat. penumbra 80% to 20% (mm)	Min. dose rate (Gy/min)	Max. dose rate (Gy/min)	Max. clinical range (mm)	Min. clinical range (mm)	Range resolution (mm)	Modulation step size (mm)	Total ocular patients (starting year)	Average ocular patients per year
1	Clatterbridge Cancer Center	Clatterbridge (United Kingdom)	62 MeV Cyclotron (low-energy)	Horizontal (DON)	DSC -SMW	Sitting	In-house	0.9	1.3	9	26	30.7	4.1	0.21	0.95	4707 (1989)	232
2	CAL-IMPT	Nice (France)	65 MeV Cyclotron (low-energy)	Horizontal (DON)	SSC -SMW	Sitting	In-house	1.0	1.4	60	80	32.2	2	0.2	0.8	7400 (1991)	244
3	UCSF Ocular Proton Therapy Center	San Francisco (USA, CA)	67,5 MeV Cyclotron (low-energy)	Horizontal (DON)	SSC -SMW	Sitting	In-house	1.0	1.5	3.5	15	30.5	7	0.1	4	2803 (1994)	80
4	Helmholtz-Zentrum Berlin and Charité (HZB/Charité)	Berlin Germany	72 MeV Cyclotron (low-energy)	Horizontal (DON)	SSC -SMW	Sitting	In-house	1.0	1.6	11	35	34	5	0.03	1	4638 (1998)	235
5	CPT PSI	Villingen (Switzerland)	250 MeV Cyclotron (high-energy)	Horizontal (DON)	DSC -SMW	Sitting	In-house	1.3	1.5	10	35	35	7.2	0.2	4	8155 (1984)	199
6	James M Slater, MD Proton Therapy and Research Center	Loma Linda (USA, CA)	250 MeV Synchrotron (high-energy)	Horizontal (DON)	SSC -SMW	Sitting	Optivus	1.5	1.2	6	10	33.5	7	0.1	n.p.	n.p. (1990)	11
7	Cyclotron Center Bronowice (IFJ PAN - Krakow)	Krankow (Poland)	230 MeV Cyclotron (high-energy)	Horizontal (DON)	SSC -SMW	Sitting	IBA + in-house	1.8	1.2	9	25	32	6	0.1	0.7	354 (2011)	30
8	Westgerman Proton Therapy Center Essen (WPE)	Essen (Germany)	230 MeV Cyclotron (high-energy)	Horizontal (DON)	SSC -SMW	Sitting	IBA	2.0	1.4	10	15	35	5	0.1	3	203 (2021)	99
9	HollandPTC	Delft (The Netherlands)	250 MeV Cyclotron (high-energy)	Horizontal (DON)	SSC -SMW	Sitting	Varian	2.0	2.0	15	15	40	6	n.p.	3	179 (2020)	49
10	Institut Curie Protontheray Center (ICPO)	Orsay (France)	230 MeV Cyclotron (high-energy)	Horizontal (DON)	SSC -SMW	Sitting	In-house	2.8	1.6	7.2	17	32	5	0.25	1	8670 (1991)	355
11	University of Florida Health Proton Therapy Institute	Jacksonville (USA, FL)	230 MeV Cyclotron (high-energy)	Horizontal (DON)	SSC -SMW	Sitting	IBA	3.0	1.2	17.5	28	34	5	0.1	3	272 (2012)	24
12	National Cancer Center Korea	Ilsan (South Korea)	230 MeV Cyclotron (high-energy)	Horizontal (DON)	SSC -SMW	Sitting	IBA	4.4	1.7	1	4	35	1	1	1	99 (2009)	7
13	Massachusetts General Hospital Proton Centers	Boston (USA, MA)	230 MeV Cyclotron (high-energy)	Horizontal (DON)	SSC -SMW	Sitting	In-house	6.0	1.5	8	12	35	8	0.1	1	2772 (2002)	117
14	Fondazione CNAO	Pavia (Italy)	228,6 MeV Synchrotron (high-energy)	Horizontal (GPN) plus ocular add-on	PBS with aperture, energy stacking	Sitting	In-house	1.5	1.5	4.5	6	32	0	0.2	0.2	458 (2016)	60
15	Shanghai Proton and Heavy Ion Center	Shanghai (China)	221 MeV Synchrotron (high-energy)	Horizontal (GPN) plus ocular add-on	PBS with aperture, energy stacking, ridge filter	Sitting	In-house	2.0	1.3	0	12	310	20	1	1^a^	81 (2018)	20
16	National Institutes for Quantum Science and Technology (NIRS)	Chiba (Japan)	140 MeV Synchrotron (high-energy)	Horizontal & vertical (GPN)	PBS, energy stacking	Supine	Toshiba	2.5	4.0	n.p.	n.p.	n.p.	n.p.	n.p.	n.p.	425 (1986)	17
17	Fred Hutchinson Cancer Center-Proton Therapy	Seattle (USA, WA)	235 MeV Cyclotron (high-energy)	Horizontal (GPN) plus ocular add-on	PBS with aperture, energy stacking, ridge filter	Sitting	IBA + in-house	3.5	1.7	20	20	75	0	0.1	0.1	408 (2015)	48
18	Northwestern Medicine Proton Center	Warrenville (USA, IL)	230 MeV Cyclotron (high-energy)	Inclined beam line (GPN)	uniform scanning, energy stacking	Sitting	IBA	3.4	2.0	1	3	320	0	1	5	370 (2011)	41
19	Mayo Clinic - Rochester	Rochester (USA, MN)	228,8 MeV Synchrotron (high-energy)	Gantry (GPN)	PBS with aperture, energy stacking	Supine	Hitachi	2.0	1.3	n.p.	n.p.	60	1	2	2	2 (2023)	2
20	New York Proton Center	New York (USA, NY)	250 MeV Cyclotron (high-energy)	Gantry (GPN)	PBS without aperture, energy stacking	Supine	Varian	4.0	9.0	n.p.	n.p.	350	41	n.p.	0	32 (2022)	8

**Abbreviation:** DON, dedicated ocular nozzle; DSC, double scattering; GPN, general purpose nozzle; n.p., information not provided; PBS, pencil beam scanning; SSC, single scattering; SMW, spinning modulator wheel.

a 1 mm, 3 mm, or 6 mm, depending if a ridge filter is used.

**Figure 1:**
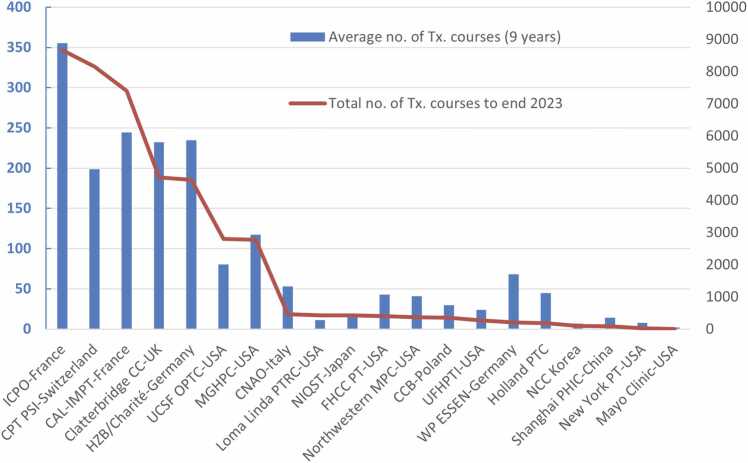
Number of ophthalmic patients treated per center. Total since center opening (line) and annual average (2015 to 2023, inclusive) (bars).

A total of 42 457 OPT treatments were reported by all participating centers at the end of 2023, including 347 helium and 367 carbon ion treatments. The accumulated total of patients treated by each center and the average number treated per year (based on the years 2015 to 2023) are presented in Figure 1. A large variation was seen in the total number of patients treated by each center (2 to 8670), with 93.8% of patients treated by 7 centers.

### Program organization

There was an almost equal split between private and publicly funded facilities, and a further 3 which identified as stand-alone research facilities (Table 2). At 11 centers, the treatment room was solely dedicated to ophthalmological treatments, and at 8 centers, ocular patients were treated in a room used also for other treatment sites.

**Table 2:** Ocular particle therapy (OPT) program organization.

**Type of facility:**	**Centers**	**Referrals:**	**Centers**

Independent or private status	8	Ophthalmologists/ophthalmology centers	20
Publicly funded or part of a university hospital	9	Self-referrals	6
Stand-alone research facility	3	**No. of referral centers:**	
		1	6
		2	3
		3	3
		4	3
		5-10	4
**Tumor board/MDT:**		**Follow-up:**	
**OPT staff participate in tumor board at:**		**Conducted by:**	
Own center	9	Ophthalmologist	19
Affiliated center	2	Radiation oncologist	14
Both own and affiliated centers	5	Other (medical oncologist, GP, radiologist)	4
Neither	4	**Conducted at:**	
		OPT center	14
		Another center	18
**Staffing model:**			
All staff share time between OPT and other work (eg, high-energy treatments)			15
Staff dedicated to OPT only			2
Mixed model: staff dedicated to OPT and staff with shared duties			3
**Full-time equivalent (FTE) staff for OPT**:		**No. patients treated annually / FTE**:	
< 1.0	2	< 10	4
1.0 to < 5.0	8	10 to < 20	6
5.0 to < 10.0	3	20 to < 30	4
10.0 to < 15.0	4	30 to < 50	2
15.0 +	3	50 +	2
*Median*	*4.75*	*Median*	*15.7*
**Activities conducted by profession:**			
**Immobilization preparation**		**Treatment planning**	
Radiographer	20	Medical Physicist	18
Medical physicist	6	Radiation Oncologist	7
Radiation oncologist	4	Dosimetrist	8
Dosimetrist	2	Radiographer	3
Nurse	1	Ophthalmologist	1
**Patient positioning for simulation**		**Plan review & approval**	
Radiographer	18	Radiation oncologist	19
Medical physicist	14	Medical physicist	18
Radiation oncologist	9	Ophthalmologist	10
Dosimetrist	4	Dosimetrist	4
Nurse	1	Radiographer	2
Engineer	1	Nurse	1
**Patient positioning for treatment**		**Daily QA**	
Radiographer	20	Medical physicist	15
Medical physicist	15	Radiographer	11
Radiation oncologist	14	Engineer	4
Dosimetrist	3	Dosimetrist	3
Nurse	1		
Engineer	1		
		**Weekly/monthly / annual QA**	
		Medical physicist	19
		Radiographer	2
		Engineer	4
		Dosimetrist	2
**Alternative therapies:**		**Additional support services:**	
Conducted at OPT center	12	Accommodation provided/funded	6
Including:		Assistance finding accommodation (not funded)	11
Plaque brachytherapy	8	Transport to and from treatment	4
External beam radiation therapy/stereotactic	8	Consultation with social worker	10
Gamma Knife	1	Consultation with financial expert	8
Surgery (including enucleation)	7	Support with cost of treatment	6
Photodynamic therapy	1	Facilitate social gatherings	4
Laser	1		
Conducted at affiliated or referral center	8		

**Abbreviations**: OPT, Ocular particle therapy; QA, Quality assurance; MDT, Multidisciplinary team.

Patients were typically referred by ophthalmologists, while 6 OPT centers received a small number of self-referrals (Table 2). The number of centers referring patients to each OPT center ranged from 1 to between 5 and 10. The staff at 16 centers participated in an ocular tumor board at their own center, an affiliated institution, or both. For 19 centers, clinical follow-up was provided by an ophthalmologist, and at 14 by a radiation oncologist or other clinician. Follow-up was mostly (n = 18) carried out at another facility, for example, the referral center, in addition to or instead of follow-up at the OPT facility.

Twelve centers offered alternative therapeutic techniques for ocular lesions at their facility, including plaque brachytherapy, external beam radiation therapy, and enucleation. For the remaining 8 facilities, alternative treatment options were available at affiliated hospitals or referral centers. Some centers provided additional supportive services, for example, accommodation, transportation, financial support or consultation with a social worker (Table 2).

At 18 centers staff divided their time between ocular treatments and other work, while the remaining 2 centers had staff dedicated to OPT. The size of the OPT team varied from 0.12 to 18 full-time equivalent (FTE)^1^ (median 4.75), and based on the average patient throughput from 2015 to 2023, the number of patients treated annually per FTE varied from three to seventy-one (median 15.7). There appeared to be no correlation between FTE and ocular patient numbers; the 2 centers with dedicated OPT staff ranked third and twelfth for total patients treated.

The preparation of immobilization aids and patient positioning were most frequently performed by radiographers (radiation therapists). Treatment planning and QA were predominantly carried out by medical physicists, although radiographers were involved in daily QA at just over half of centers. Plan review and approval was conducted by radiation oncologists and medical physicists at most centers (n = 18), with ten obtaining input from the ophthalmologist for plan review and approval.

### Patient population and prescription

The most common lesions were choroidal and/or ciliary-body melanomas, followed by iris melanomas, hemangiomas, choroidal metastases, conjunctival tumors, and angiomas. Other conditions, including age-related macular degeneration and retinoblastoma, were treated by 3 and 2 centers, respectively (Table 3).

**Table 3:** Tumor types and fractionation schemes.

Tumor type (no. of centers treating this type):	Dose and fractionation schemes (no. of centers):
Choroidal melanomas (19)	60 Gy (RBE) / 4fr (9)
	70 Gy (RBE) / 5fr (2)
	70 Gy (RBE) / 5fr or 50 Gy (RBE) / 5fr (1)
	60 Gy (RBE) / 4fr or 56 Gy (RBE) / 4fr (1)
	50 Gy (RBE) / 5fr (2)
	56 Gy (RBE) / 4fr (1)
	57.2 Gy (RBE) / 4fr (1)
	68 Gy (RBE) / 4fr (1)
	45 Gy (RBE) / 5fr (1)
Iris melanomas (12)	60 Gy (RBE) / 4fr (6)
	50 Gy (RBE) / 5fr (1)
	50 Gy (RBE) / 4fr (1)
	70 Gy (RBE) / 5fr (2)
	56 Gy (RBE) / 4fr (1)
	57.2 Gy (RBE) / 4 fr (1)
Conjunctival tumors (8)	60 Gy (RBE) / 4fr (2)
	60 Gy (RBE) / 8fr (1)
	57.2 Gy (RBE) / 4fr (1)
	52 Gy (RBE) / 4fr (1)
	70 Gy (RBE) / 5fr (1)
	56 Gy (RBE) / 4 f or 40 Gy (RBE) / 4fr (1)
	60 Gy (RBE) / 4fr or 48 Gy (RBE) / 4fr (1)
Choroidal haemangiomas (11)	20 Gy (RBE) / 4fr (4)
	20 Gy (RBE) / 8fr (2)
	20 Gy (RBE) / 4fr or 20 Gy (RBE) / 10fr (1)
	12-20 Gy (RBE) / 4fr (1)
	15 Gy (RBE) / 4fr (1)
	18-22 Gy (RBE) / 4 fr (1)
	19.8 Gy (RBE) / 4fr (1)
Angioma (7)	19.8 Gy (RBE) / 4fr (1)
	18-20 Gy (RBE) / 4fr (1)
	20 Gy (RBE) / 4fr (2)
	35 Gy (RBE) / 4fr (1)
	20 Gy (RBE) / 4fr or 20 Gy (RBE) / 10fr (1)
	20 Gy (RBE) / 8fr or 60 Gy (RBE)/ 4fr (1)
Choroidal metastases (8)^a^	40 Gy (RBE) / 4fr (1)
	20-40 Gy (RBE) / 4fr (1)
	60 Gy (RBE) / 4fr (1)
	20 Gy (RBE) / 2fr or 22-28 Gy (RBE) / 2fr (1)
	24 Gy (RBE) / 2fr (1)
	48 Gy (RBE) / 12fr (1)
	50 Gy (RBE) / 5fr (1)
Macular degeneration (3)^b^	16-20 Gy (RBE) / 4fr (1)
	14 Gy (RBE) / 1fr (1)
Retinoblastoma (3)	31.6 Gy (RBE) / 6r (1)
	45 Gy (RBE) / 25fr (1)
	50 Gy (RBE) / 25fr (1)
Medulloepithelioma (1)	50 Gy (RBE) / 4fr (1)

a One center did not provide the dose and fractionation scheme.

b One center did not provide the dose and fractionation scheme.

There was variation in treatment dose and fractionation among the centers (Table 3). The most common prescription was 60 Gy (RBE) in four fractions to treat choroidal and ciliary body melanomas (n = 9), followed by 70 (n = 2) or 50 (n = 2) Gy (RBE) in five fractions. At one center, the standard dose regimen was reduced from 70 Gy (RBE) to 50 Gy (RBE) in 5 fractions for small tumors near the optic disc and macula. The protocol for conjunctival treatments ranged from 40 to 60 Gy (RBE) in four or five fractions, with a variance of one center delivering 60 Gy (RBE) in 8 fractions. For benign conditions, such as hemangiomas and angiomas, most centers used a total dose of 15–20 Gy (RBE) over 4 fractions, with 2 centers treating with the same dose over 8 fractions.

A small subset of patients treated with OPT for ocular conditions were children. Nine centers reported treating 1 to 5 children per year, with no clear age limit (as young as 2 months, and 9 years on average), for conditions such as choroidal hemangiomas, choroidal and ciliary body melanomas or retinoblastomas. Eight of the centers treating pediatric patients also reported the largest total treatments and annual throughput.

### Proton (particle) therapy system overview

Table 1 gives an overview of the twenty operational OPT centers, their system setup (eg, accelerator type and energy; nozzle type), and beam parameters for OPT treatment. OPT systems at nine institutions were developed and built in-house and two were a combination of vendor and in-house developments. Five different companies developed the other nine installations.

The systems were divided into 4 groups^2^: low-energy machines (< 75 MeV) using a dedicated horizontal ocular nozzle (LEDON) (n = 4); high-energy machines (> 120 MeV) using a dedicated horizontal ocular nozzle (HEDON) (n = 9); high-energy machines using a fixed general-purpose nozzle (HEGPN) (n = 5) and high-energy machines using a gantry (n = 2). Three centers with a general-purpose nozzle used a special in-house developed adapter for ocular treatments. Four of the HEDON systems were developed in-house, and the other five were vendor-based. The LEDON systems were all in-house developments.

All seven centers that started ophthalmic treatments after the last survey in 2015 were high-energy systems (> 120 MeV). Of these 7 centers, 5 had horizontal beamlines (2 with a dedicated ocular nozzle and three general-purpose nozzles with ocular adapters), and 2 were gantry-based systems with general-purpose nozzles. The cumulative number of OPT treatments reported by center type (Table 4), shows that although dominated by dedicated systems (LEDON and HEDON), there was an increase in the share carried out by HEGPN and gantry systems from 2015 to 2023. Although cumulative totals indicate a predominance of high-energy treatments, accounting for the closure of low-energy lines (eg, Paul Scherrer Institute and TRIUMF) shifts the balance toward low-energy treatments. Nonetheless, the proportion of high-energy treatments continues to increase.

**Table 4:** Beam characteristics and interlocks.

**Summary of beam characteristics**						
System type (number of centers)			LEDON (4)	HEDON (9)	HEGPN (5)	Gantry (2)

Percentage (rounded) of patient courses:						
2015			51	46	1	2
2023			43	46	6	6
Cumulative total^a^ (%)			19548 (46)	21133 (50)	964 (2)	812 (2)
Distal fall-off (mm water equivalent)		Average	∼ 1	2.9	2.6	3.0
		Range	0.9-1.0	1.3-6.0	1.5-3.5	2.0-4.0
Lateral penumbra (mm water equivalent)		Average	1.5	1.5	2.1	5.2
		Range	1.3-1.6	1.2- 2.0	1.3 – 4.0	1.3-9.0
Max. dose rate for typical clinical beams (Gy/min)		Average	39	17.9	10.3^b^	n.p.
		Range	15-80	4-35	3-20	n.p.
**Water equivalent range and modulation (mm)**						
			Average	Median	Range	
Clinical range		Max.	38	34	30.5-350	
		Min.	6.9	5.0	0-41	
Range resolution			0.4	0.2	0.03-2	
Modulation step size			1.8	1.0	0-5	

**Abbreviations**: HEDON, high-energy machines (> 120 MeV) using a dedicated horizontal ocular nozzle; HEGPN, high-energy machines using a fixed general-purpose nozzle; LEDON, low-energy machines (< 75 MeV) using a dedicated horizontal ocular nozzle; n.p. - not provided.

a The HEDON total includes 5300 patients treated with the 72 MeV proton beam at The Paul Scherrer Institute (PSI) prior to 2010. Considering treatment numbers from PSI (72 MeV), the Harvard Cyclotron (160 MeV), and TRIUMF (low energy) beamlines, which have closed, the proportion of LEDON and HEDON becomes 55% and 41%, respectively.

b Based on data from 4 HEGPN centers.

### Beam delivery and characteristics

All centers with a dedicated ocular nozzle (LEDON and HEDON) used a combination of single- or double-scattering system and a spinning modulator wheel to form a uniform irradiation field with patient specific aperture. Other centers relied on scanning approaches, either uniform scanning or pencil beam scanning with or without aperture (Table 1).

Beam parameters for the 4 system types are summarized in Table 4. LEDON systems had the steepest distal fall-off (∼1.0 mm) combined with steep lateral penumbrae (average: 1.5 mm) and the highest dose rates. These parameters were almost matched by well-developed HEDON systems, for example, at the Paul Scherrer Institute,^12^, ^13^, ^14^ but overall, 3 was a wider spread in distal fall-off (range: 1.3 to 6.0 mm), and maximum reported dose rates for typical clinical beams were lower on average. The five HEGPN systems had less spread in distal fall-off (range: 1.5 to 3.5 mm), larger lateral penumbrae (average: 2.1 mm), and low dose rates. The gantry-based systems reported no information on dose rate, large distal fall-off (average: 3.0 mm), and the widest range for lateral penumbrae (1.3 to 9.0 mm).

The reported maximum water equivalent clinical ranges varied from 30.5 to 350 mm (Table 4). Depending on the system, the range could be chosen in steps from 0.03 to 2 mm whilst the modulation of the spread-out Bragg Peak was noted to be in steps of 1.8 mm on average.

Thirteen installations had an interlock system for beam symmetry or flatness, and at six installations, the patient collimator was barcode verified. Eight centers had servo systems to correct dose (n = 5), symmetry (n = 5), or flatness (n = 3), including 2 with automatic correction of all 3 parameters.

### Treatment planning workflow and systems

Table 5 lists the treatment planning systems (TPS) employed by OPT facilities with LEDON, HEDON, HEGPN, and gantry-based systems, subdivided into dedicated ocular or general-purpose TPS. A dedicated ocular TPS was utilized by all centers with a dedicated eye nozzle, ie, LEDON and HEDON systems (n = 13). Over half of these centers used EyePlan (n = 8), followed by the Eclipse Ocular Proton Planning system (Varian, Palo Alto, California, USA) (n = 3), Octopus (n = 1), and RayOcular (RaySearch Laboratories, Sweden) (n = 1). General-purpose TPS were mostly utilized by centers with a general-purpose nozzle for OPT treatment ie, HEGPN and gantry-based systems (n = 7), although for one center (HEGPN system with ocular adapter), the Eclipse Ocular Proton Planning was the TPS in use.

**Table 5:** Systems and practice patterns for treatment planning and 3D imaging techniques.

Treatment planning systems and 3D imaging techniques		Number of centers
TPS: LEDON systems	EyePlan^d^	3
	Octopus^d^	1
TPS: HEDON systems	EyePlan^d^	5
	Eclipse Ocular Proton Planning^d^	3
	RayOcular^d^	1
TPS: HEGPN systems	Eclipse Ocular Proton Planning^d^	1
	Siemens Syngo	1
	XiDose (in-house system supported by Elekta)	1
	RayStation general purpose	1
	CMS Xio	1
TPS: gantry-based systems	Eclipse general purpose	2*
	* Matlab (SOBP creation), in-house GPU MC (dose calculation) used by 1 center	
CT-scan: treatment planning	All ocular patients receive a CT scan	10
	Some ocular patients receive a CT scan	4
	CT scan is not performed for ocular patients	6
	CT slice thickness (0.5 - 0.625 mm)	8
	CT slice thickness (0.75 - 1.25 mm)	6
CT-scan: uses	Clip positions w.r.t eye anatomy	12
	Eye model dimensions	6
	Tumor geometry	5
	Delineation/dose estimation of OARs (eg lacrimal)	6
	Screen for extra-ocular extension	2
	Dose calculation	6
CT-scan: gaze control	Fixation light or aid- variable gaze	6
	Fixation light/ aid/ verbal coaching- neutral gaze	6
	No gaze control, arbitrary position	2
MR scan: treatment planning	All ocular patients receive an MRI scan	6
	Some ocular patients receive an MRI scan	6
	MRI scan is not performed for ocular patients	7
MR scan: uses	Eye geometry	8
	Clip-tumor distances	4
	Tumor height/base dimensions/shape	11 / 9 / 12
	Delineation of intra / extra-ocular OARs	8 / 6
	Screen for extra-ocular extension	7
MR scan: gaze control	Fixation light or aid- variable gaze	2
	Fixation light/ aid/ verbal coaching- neutral gaze	5
	No gaze control-arbitrary position	4
MR scan: field strength	1.5 T	5
	3.0 T	5
	Both 1.5 T and 3.0 T	1
	0.23 T	1
MR scan: coil type	Head	8
	Eye-specific / surface coil / loop coil	4
Treatment plan preparation time *(excluding marker surgery and patient-specific QA)*	< 1 day	3
	1 - <2 days	3
	2 - <3 days	3
	3 - <4 days	3
	4 - <5 days	3
	5 days or more	5

‘d′ indicates dedicated ocular TPS.

Abbreviations: HEDON, high-energy machines (> 120 MeV) using a dedicated horizontal ocular nozzle; HEGPN, high-energy machines using a fixed general-purpose nozzle; LEDON, low-energy machines (< 75 MeV) using a dedicated horizontal ocular nozzle; QA, Quality assurance; SOBP, spread-out-Bragg-peak; TPS, treatment planning system.

Over the last decade, significant progress has been made in integrating advanced 3D imaging techniques into ocular treatment planning workflows alongside traditional planar images. According to the survey findings (Table 5), 14 centers routinely incorporated CT data, while twelve utilized MRI information for planning purposes. Ten centers conducted a CT scan, and 6 centers conducted an MRI scan for each ophthalmic patient as part of their standard procedure. Depending on the TPS system in use, 3D imaging data was used directly in the TPS or indirectly to support treatment planning. For example, 5 EyePlan users reported the use of CT and/or MRI to inform treatment planning, even though the TPS itself does not support the integration of 3D image data.

The level of integration of CT imaging into the clinical workflow is shown in (Table 5). CT was used for determination and/or verification of the eye model, tumor geometry, and fiducial marker (clip) positions relative to eye anatomy regardless of type of TPS used. In addition, for general-purpose TPS, the CT image data was utilized for dose calculation and delineation of dose estimation to extra-ocular structures. Five centers employed specialized commercially available reconstruction algorithms, such as iMAR or OMAR for correcting metal artifacts.

MRI was most frequently used for defining tumor apex height, base dimensions and shape and to screen for extra-ocular extension (Table 5). Additional uses include determination of clip-tumor distances, eye geometry, and delineation of intra-ocular and extra-ocular organs at risk (OAR). Equal use of 1.5 T and 3 T MRIs was employed, and one facility used a 0.23 T orbital MRI. Only 4 centers were equipped with dedicated eye coils.

For target delineation, centers using a dedicated ocular TPS (n = 14) generated a geometric tumor model based on modeled clip positioning, fundus photography, and caliper measurements. Ultrasound (n = 17) and fundus photography (n = 18) were routinely used to estimate tumor-related parameters such as apex height, base dimensions and tumor shape, and sometimes for delineation of intra-ocular OARs, screening for extra ocular extension or to obtain clip-tumor distances. Several centers used optical coherence tomography (n = 7) and fluorescence angiography (n = 8) for similar assessments. Centers using a general-purpose TPS relied more heavily on CT and MRI images for target and OAR delineation.

CT (n = 13) and MRI (n = 12) scans were mainly acquired at the OPT center, with some from external centers (CT n = 2, MRI n = 5). In contrast, ultrasound (n = 15), fundus images (n = 16), and surgical measurements (n = 12) were typically sourced externally, with fewer obtained locally (ultrasound n = 6, fundoscopy n = 6, surgical data n = 5). Some centers reported a combination of externally and internally acquired data.

Wedges were used by 5 centers for some patients; 5 centers used compensators, and 1 used both. The number of cases treated with compensators or wedges varied significantly among the centers that used these beam-modifying devices. Twelve centers planned and treated some cases with the beam passing through the eyelid,^15^ taking account of the lid tissue in the treatment plan with a simple eyelid model (dedicated ocular TPS) or density override in the CT scan (general-purpose TPS). Reasons for planning and treating through eyelid tissue included large treatment fields, superior tumor location, difficulty in fully retracting lids, discomfort or pain caused by lid retraction, avoidance of treating the lid margin to not cause eyelash loss (madarosis), multifield planning approach (using gantry-based systems), and conjunctival treatments, where eyelid tissue was included in the target.

Centers estimated that treatment preparation per patient, excluding fiducial marker surgery and QA, required less than one to over five days (Table 5). Centers with higher patient throughput (over 2000) reported the quickest times (< 3 days). Preparation time appeared to be linked more to center experience than to planning system or use of 3D-imaging.

### Patient setup and immobilization

Ophthalmic treatments were delivered in the seated position at most facilities (n = 17), >95% of patients treated in 2023. Notably, all facilities that participated in the previous survey only offered seated treatments.^3^ Among the newer facilities, opened since 2015, only three performed seated set-up only, while two could accommodate seated and supine, and two offered supine set-up only. Treatment chairs were produced on custom order by multiple manufacturers, but none of these are currently offered commercially.

All facilities used thermoplastic masks for immobilization, and 14 reported using a bite block in addition to the mask. Restraining straps for the head and chin rest were also used by some centers to aid immobilization.

Sixteen centers retracted eyelids out of the field, including all passive-scattered lines with a dedicated ocular nozzle (LEDON and HEDON). Most facilities (n = 13) reported using one or two larger retractors (one per lid); 6 facilities used multiple small retractors per lid, 4 centers sometimes used tape as an alternative, while one used tape as the sole retraction method. Fourteen facilities reported the use of light-field projection or on-axis camera to check eyelid position relative to the treatment field. All centers reported that treatments were sometimes delivered with eyelid margins (rims) in the field, when full retraction was not possible. Mitigations to minimize side effects included recording and varying lid position (n = 3). Mitigations to minimize range effects included: applying density overrides (on CT image data) to assess the effect of eyelid position variation, applying range and modulation adjustments based on actual eyelid position and thickness, and the use of multiple fields.

### Patient alignment and online monitoring

Nineteen centers reported the use of orthogonal x-ray pairs for treatment setup. This was complemented with gantry-mounted cone-beam CT at one of the newer facilities. Eleven delivered some treatments without the use of fiducial markers for alignment: mostly anterior (eg, iris, ciliary body, conjunctival) melanomas, metastases, and benign conditions (eg, hemangiomas). Macular degeneration, very posterior tumors, and medical comorbidities were also mentioned as candidates for marker-less treatments. One HEGPN facility indicated that it presently treats all uveal melanomas without fiducials.

Various systems are used for patient position verification, these were split between three categories: in-house developed (n = 5), TPS-based, such as EyePlan (n = 5) and RayOcular (n = 1), or provided by machine vendors (n = 8). Nineteen facilities applied translational adjustments for setup, fifteen reported additional adjustments like pitch, roll, or yaw on the patient positioner, and nine applied rotational corrections to the collimator.

All centers except one reported using cameras to monitor eye position during treatment delivery. Manual gating was the most common method; 2 facilities have implemented automated gating. Post-treatment confirmation imaging was performed at 5 facilities.

Treatment time slots ranged from 10–14 min (n = 1), 20–29 min (n = 7), 30–45 min (n = 7), to over 45 min (n = 5). For only 4 centers, the scheduled duration included time allocated for QA, which varied between 5 and 45 minutes. No correlation was observed between the reported treatment time slots and either the number of patients treated or the inclusion of QA within the allotted time.

### Dosimetry and quality assurance

All participating centers followed the IAEA TRS-398 Code of Practice^16^ for reference dose determination, except for one center which followed the Japanese Standard Dosimetry of Absorbed Dose to Water in External Beam Radiotherapy.^17^ Sixteen centers reported using water as the medium for reference dosimetry with a traceable ion chamber (Table 6), three of those centers reported using Polymethyl methacrylate (PMMA) or solid water in addition to water, while three centers used PMMA phantoms only. Eight centers employed plane parallel ionization chambers, 6 centers used cylindrical ionization chambers, while the remaining 6 centers employed both types. The most frequently used chamber was the PTW Advanced Markus, followed by the PTW Semiflex 0.125cc, Semiflex 0.3 cc, and IBA PPC05 chambers. A handful of centers report the use of alternative small-volume thimble chambers (IBA CC-13, Exradin Miniature Shonka or PTW 30013 Farmer-type).

**Table 6:** Dosimetry and quality assurance.

**Reference dosimetry:**			
**Code of practice:**	**No. of centers**	**Ionization chamber type:**	**No. of centers**

IAEA TRS-398	19	Plane-parallel	8
Japanese Standard Dosimetry of Absorbed Dose to Water in External Beam Radiotherapy	1	Cylindrical	6
		Plane-parallel & cylindrical	6
**Phantom material:**		Ionization chamber model:	
Water only	13	PTW Advanced Markus	10
Water & PMMA or solid water	3	PTW Semiflex 0.125cc	5
PMMA only	3	PTW Semiflex 0.3cc	4
		IBA PPC05	4
		IBA CC-13, Exradin Miniature Shonka, PTW 30013	4
**Patient-specific dosimetry / MU determination:**		**Patient-specific aperture check:**	
Measured with a traceable ionization chamber…		Overlay on printout from the TPS	13
…in water with patient aperture	2	X-ray of aperture (without patient present)	6
…in water without patient aperture	3	Inspection of light-field (without patient present)	2
…in plastic with patient aperture	3	Measurement of dose profile behind aperture	1
…in plastic without patient aperture	6		
Analytical model or table	6		
Treatment planning system	2		
Monte Carlo	2		
**Range, modulation or SOBP measurement:**		**Lateral dose profile measurement:**	
Parallel-plate ionization chamber in water	12	Gafchromic film	10
Parallel-plate ionization chamber and Perspex wheel	3	Radiographic film	3
Multilayer ionization chamber	5	Scanning diode	6
Diode and water tank	3	Scintillating detector	6
Diode and Perspex wheel	3	Scanning ionization chamber	3
Parallel plate in plastic phantom	2	Diamond detectors	2

**Abbreviations:** MU, monitor units; QA, Quality assurance; SOBP, spread-out-Bragg-peak; TPS, treatment planning system.

**Figure 2:**
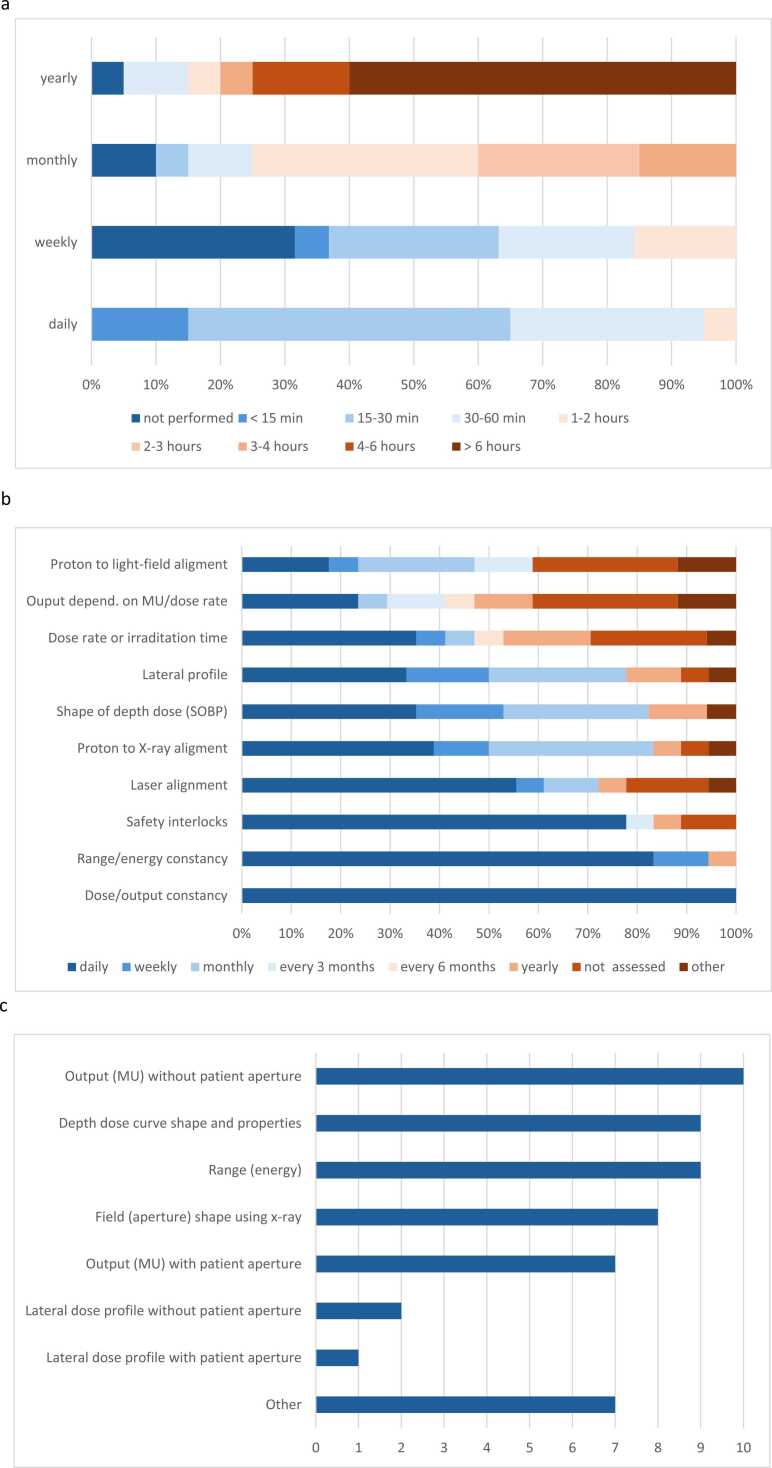

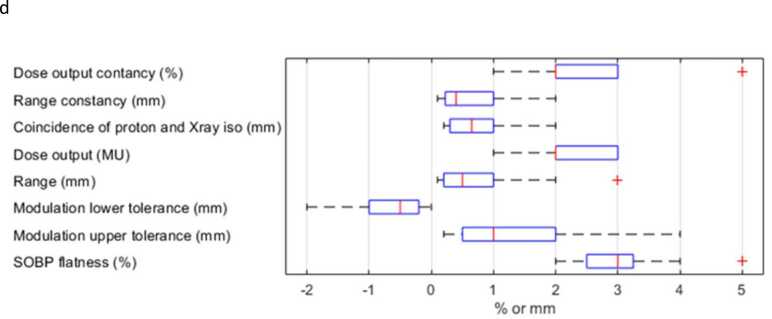
Quality assurance (QA) in OPT; (a) Time for machine QA (answered by 20 centers); (b) Frequency of check of individual parameters (answered by 18 centers); (c) Dosimetric parameters checked during patient QA (answered by 18 centers); (d) Box plots of tolerances (answered by 17 centers). Median values (red line) and outliers (red cross) are indicated. Blue boxes highlight the range of values from most centers. Abbreviation: MU, monitor units; OPT, ocular particle therapy; SOBP, spread-out-Bragg-peak.

Variation in the QA schedule was noted between centers (Figure 2). Figure 2a indicates the time taken for daily, weekly, monthly, and annual machine QA checks at the 20 operational centers. More than half (n = 13) spent less than 30 minutes on daily checks. Most spent 15 minutes to 2 hours on weekly tests, monthly checks took between 1 and 3 hours at most facilities (n = 15), while annual checks required more than 6 hours at 12 centers. For each new treatment course, the time taken for patient-specific QA varied from less than 15 minutes (n = 4), 15 to 30 minutes (n = 6), 30 to 60 minutes (n = 7), to more than 1 hour (n = 3).

Figure 2b shows the frequency of checks for individual parameters considered to be most important for ocular PT systems. They may be divided into those relating to the machine and beamline, and those related to patient set-up and imaging. Machine-related parameters checked most frequently were dose (output) constancy, range (energy) constancy and safety interlocks, followed by spread-out-Bragg-peak (SOBP) shape and lateral beam profile. The most frequently performed treatment precision and positioning parameters were laser alignment and proton x-ray isocenter coincidence checks, followed by proton and light-field coincidence checks. The following patient-specific parameters were checked most frequently (Figure 2c): output and monitor units (MU) with or without patient aperture, range (energy), and shape of depth dose distribution.

A variety of methods were reported to determine the output and MU for a patient-specific treatment field (Table 6); the most common method used a dose calibration performed with a traceable ion chamber. The output was measured either in water or plastic with or without a patient aperture installed. Six centers used an analytical model or table to determine MU, 2 used the TPS, and 2 reported using Monte Carlo for MU calculation. Six centers used 2 different methods, and 1 reported using 3. The Relative Biological Effectiveness (RBE) correction factor of 1.1 was universally used for proton dose, with most centers (n = 17) applying this factor to convert from measured to clinical dose. Both carbon ion centers used their TPS to determine the RBE-weighted dose distribution, one specified use of the LEM1 based model.

Patient-specific aperture shape was checked in different ways: overlaying on a printout from the TPS was the most common method, followed by x-ray of the aperture or inspection of light field without the patient present (Table 6). One center measured the dose profile behind the aperture, and more than one method was used by five centers. All centers using wedges (n = 5) compared the position and orientation to a printout from the TPS, and all centers using compensators (n = 3) checked the thickness at select points using a height gauge.

Range, modulation, and SOBP shape were measured using the following equipment: a parallel-plate ionization chamber was most often used either in a water tank or in combination with a Perspex wheel (Table 6). The use of a multilayer ionization chamber, a diode in combination with a Perspex wheel and parallel plates in a plastic phantom was also reported. Five centers used 2 of the methods described. Most devices used for SOBP measurement were developed in-house (n = 12). For the measurement of lateral dose profiles most centers utilized Gafchromic™ film, followed by scanning diodes or scintillating detectors. Some centers reported the use of scanning ionization chambers, radiographic films, and diamond detectors (n = 2). Four centers used 2 different methods, and three centers used 3 different methods for this task.

Tolerances for the above procedures are shown in Figure 2d. The box charts indicate consistency between centers in tolerances for beam characteristics such as range, range constancy, minimum modulation (SOBP), and SOBP ‘flatness’, but more variation in tolerances for maximum modulation. The tolerances for x-ray isocenter coincidence checks varied between 0.3 to 1 mm for most centers, with one outlier at 2 mm.

### New developments and outlook

Most operational centers (n = 19) expected to continue treating patients with their existing configurations. Nine centers had either ongoing upgrades or plans to upgrade their beamlines within 3 months to 5 years. Among them, 6 centers reported modification of existing beamlines, while three had plans to implement entirely new ones.

Sixteen centers reported plans to upgrade their ocular TPS; most (n = 9) were intending to incorporate RayOcular^6^ and for some (n = 5) this was to address the obsolescence of their current system. The RayAnatomy component of RayOcular, with functions to contour targets based upon funduscopic imaging is of interest as many centers transition to pencil beam based planning on RayStation. Other topics being examined include integration of MRI into treatment planning, exploration of FLASH, and marker-free image-based ocular treatment.

### Comparison with the 2015 survey

Table 7 highlights key differences between this and the previous survey. The cumulative treatment total and annual treatment numbers have increased, demonstrating growth, and this is accompanied by a shift towards decentralization. Currently, 11 centers perform 95% of treatments, up from 7 nearly a decade ago.

**Table 7:** Key differences between current and 2015 surveys.

	2015 Survey	Current Survey
No. of participating centers	10 operational	20 operational (plus 3 with plans to become operational, 23 total)
Total OPT treatments reported	28,891 (to end 2014)	42 457^a^ (to end 2023)
Annual OPT treatment numbers	1536 (in 2014)	1942 (in 2023)
Number of centers performing 95% of treatments	7	11
No. of multiroom facilities (%)	5 (50%)	16 (80%)
No. of centers with low-energy accelerator (%)	5 (50%)	4 (20%)
Beamline orientation	10 (100%) horizontal	16 (80%) horizontal 1 (5%) horizontal & vertical 1 (5%) inclined 2 (10%) gantries
Nozzle type	10 (100%) dedicated ocular	13 (65%) dedicated ocular 7 (35%) general purpose
Accelerator type	10 (100%) cyclotrons	15 (75%) cyclotrons 5 (25%) synchrotrons
Delivery technique	10 (100%) scattering 8 (80%) single, 2 (20%) double scattering	13 (65%) scattering 11 (55%) single, 2 (10%) double scattering 7 (35%) scanning 2 (10%) uniform scanning (wobbling) 1 (5%) pencil beam scanning 4 (20%) pencil beam scanning with aperture
Depth (energy) modulation	10 (100%) spinning wheel	11 (55%) spinning wheel 7 (35%) energy stacking (2 not provided)
Ocular system developed in-house or by vendor:	9 (90%) in-house 1 (10%) by vendor	9 (45%) in-house 9 (45%) by vendor 2 (10%) vendor plus in-house
First line technical support	9 (90%) in-house	10 (50%) in-house 7 (35%) vendor technical support on-site
Treatment planning system (TPS)	10 (100%) dedicated ocular	14 (70%) dedicated ocular 6 (30%) general purpose
No. of centers using a TPS which support 3D images	1 (10%)	8 (40%)
No. of centers using MRI	4 (40%)	12 (60%)
No. of centers using CT	5 (50%)	14 (70%)
No. of centers using wedges; compensators	7 (70%); 2 (20%)	5 (31%); 5 (31%)
Follow-up carried out by radiation oncologist	3 (30%)	14 (70%)
Quality assurance procedures carried out by radiation therapists and technical staff	3 (30%)	14 (70%)

**Abbreviation:** OPT, ocular particle therapy.

a The 2015 survey cumulative total includes 2973 treatments at The Harvard Cyclotron reported by the Massachusetts General Hospital (MGH). The current survey total does not include Harvard patients; MGH reported the cumulative number treated at the hospital proton center since 2002.

Other notable developments include a rise in multiroom facilities and an increased prevalence of vendor-based systems, often supported by vendor-provided technical assistance. There has been a shift towards the use of high-energy, general-purpose beamlines and gantry-based systems for ocular treatments, moving away from the previous dominance of dedicated low-energy systems. Additionally, the adoption of advanced imaging modalities, such as CT and MRI, has grown, with more centers now integrating these technologies into their planning workflows. This evolution is accompanied by a transition towards general-purpose TPSs, further reflecting the dynamic changes in clinical practice.

Professional roles have evolved, with radiation oncologists now increasingly involved in follow-up care of eye patients, and radiation therapists and technical staff assuming expanded responsibilities in QA, patient positioning, and treatment delivery.

## Discussion

This study presents updates from an international survey covering OPT activities at 20 centers from 2015 to 2023, doubling the number of centers previously reported.^3^ The number of treated patients increased by nearly 50%, reflecting the field’s growth. Nonetheless, patient care remains concentrated in a limited number of highly specialized centers, indicating the centralization of expertise in ocular oncology.

While proton therapy remains the standard treatment modality for most ocular tumors, several centers have explored the use of carbon and helium ions, investigating these particles for their potential radiobiological advantage. Most centers also offer alternative treatments such as brachytherapy, external beam radiation therapy with photons, or surgery, reflecting an integrated and multidisciplinary approach for optimal care, without bias. Dedicated tumor boards are utilized by many centers, which support collaborative clinical decision-making.

Referral patterns continue to be led by ophthalmologists, with limited self-referrals. Ophthalmologists are also involved in patient follow-up at all centers, based on the necessity for specialized post-treatment evaluation. Radiation oncologists contribute to follow-up at most centers, although their involvement is often secondary due to the specific nature of ophthalmologic assessments. A combined approach (by ophthalmologist and radiation oncologist) is often and increasingly adopted, providing the best scenario for a comprehensive follow-up.

There has been a shift in the organizational structure of centers performing OPT treatments, with equal representation of public and private institutions, a notable change from prior years, with more public centers. Shared staffing models with the main radiation therapy department are common, highlighting the need for balancing specialized training with resource availability. Team responsibilities resemble those in conventional radiation therapy, though additional competencies are required due to the unique technical demands of OPT treatments.

The spectrum of ocular tumor diagnoses for treatment remains stable, with uveal melanomas as the predominant indication. The doubling of patients treated over the years, compared with other treatment options for this disease, reflects the favorability of patient treatment experience and outcomes and thus the wider importance of OPT programs. Treatment fractionation has also remained consistent (4 or 5 fractions for most tumors), although variability exists for rarer sites (eg, conjunctival tumors), underscoring the need for standardized guidelines.

Since the last survey, new centers have adapted high-energy systems, with beam degraders in non-dedicated lines, for ocular use, indicating a trend toward leveraging existing infrastructure for broader treatment capabilities. Nonetheless, dedicated low-energy systems still account for 20% of the operating centers, having superior dosimetric parameters (eg, sharp distal fall-off, steep lateral penumbrae) and, accounting for closed facilities, a larger number of cumulated patients with long clinical experience and satisfactory follow-up. General-purpose systems offer wider accessibility but may compromise on beam sharpness,^18^ highlighting a trade-off between technical optimization and treatment availability. Most centers continue to rely on passive-scattering techniques, although scanning techniques, including pencil beam scanning, some on gantry-based systems, are gaining ground, especially at newer installations. In-house developed systems are still in use but are reducing proportionally due to the availability of high-energy gantry beamlines and regulatory requirements. The clinical impact of these new approaches is still under evaluation. For some older centers, faced with the challenge of aging and obsolescent systems, the most practical way to secure OPT service longevity may be to integrate the ocular service into a mainstream PT program, adapting the general-purpose system for ocular work. While this solution may not achieve the dosimetric quality of dedicated OPT systems, it would allow the continuation of clinical expertise and established referral patterns. It is expected that OPT development will continue at some of the older dedicated eyeline systems, by update and refurbishment, in parallel to the adaptation of general-purpose systems.

The variability in system design has led to heterogeneous QA practices. QA schedules and tolerances differ with system characteristics and usage, with most centers following similar beam and treatment verification principles despite these differences. The lack of standardization across beamlines contrasts with the relative uniformity in conventional radiation therapy.

TPS vary widely. Dedicated ocular systems dominate in older centers and typically rely on geometric modeling and fundus photography, while newer centers use multipurpose or image-integrated systems. Although the number of centers using a geometrical versus image-based approach is comparable, most ocular treatments to date have been planned with the geometrical model approach because centers using a dedicated TPS typically have the largest patient throughput.

The increased use of 3D imaging (CT and MRI) supports more precise modeling and dose calculation. TPS upgrades are a priority, particularly as some legacy systems are no longer supported. New systems offer improved modeling and dose calculation capabilities, though some still lack specific features critical to ocular treatments and there are potential problems for eye positional changes between the CT imaging for planning and the treatment setup stages. Further development and shared clinical experience will be essential for smooth adoption and optimization.

Most centers treat patients in a seated position and use eyelid retractors, though methods differ. All centers employ orthogonal x-ray imaging for setup verification and visual monitoring during treatment delivery, with limited adoption of CBCT. There is variation in systems, with at least half utilizing TPS-based or in-house solutions for treatment set-up. The absence of dedicated commercial systems fulfilling the unique requirements for eye position verification presents a challenge, especially for centers transitioning from a TPS with integrated position verification. A growing number of centers treat without fiducial markers, particularly for anterior tumors, the accuracy of which relies on real-time surface imaging.

Standard dosimetry protocols (IAEA TRS-398) and the use of similar detectors are widely adopted, with water-based reference measurements common. QA tasks are time-intensive, particularly daily and annual checks, though integration with general-purpose systems may drive efforts to streamline these processes.

Monitor unit calibration and dose verification vary, with some centers still relying on direct time-consuming measurement, while others have adopted or are evolving towards analytical or Monte Carlo-based models. These analytical models can help centers to correct dosimetric readouts for non-ideal combinations of ionization chamber size and field size. Field verification techniques differ across institutions, reflecting both legacy in-house developed systems and innovations in workflow optimization.

Despite the historic centrality of ocular therapy in the evolution of PT, its relative presence has diminished, with only a minority of the new centers currently offering these treatments. Nevertheless, the field remains poised for growth, driven by increasing clinical interest, published results, emerging technologies, and broader dissemination of expertise.

Key challenges include the obsolescence of dedicated TPS, the absence of commercial low-energy accelerators, and the integration of ocular treatments into non-dedicated systems. Additionally, emerging paradigms such as FLASH therapy, mini beams, in combination with immunotherapy^19^ or targeted agents, represent future directions that demand rigorous clinical evaluation.

Advancing the field will require robust clinical trials, improved technology integration, and continued collaboration across disciplines to ensure precise, effective, and accessible care for ocular tumor patients.

This survey represents the majority of OPT centers, 20 of 23 known to be in operation at the end of 2023, ensuring that the results accurately reflect practices in OPT globally. Although 20 centers participated, 1 center provided only a partial response. The results are therefore reported in terms of the number of centers rather than percentages, which could be misleading. Although the survey did not capture ocular treatment numbers from non-participating centers and does not account for beamlines that have closed, treatment numbers at TRIUMF to 2017 (n = 204), the Harvard Cyclotron to 2002 (n = 2973), and the 72 MeV proton beam at Paul Scherrer Institute to 2010 (n = 5300) were considered.

To minimize misinterpretation of survey questions and reduce bias, the survey was designed and results interpreted by medical physicists and clinicians from 18 institutions, representing a wide range of systems, workflows, and experience. The accuracy of the survey data depends on the participants’ responses. Any data queries, discrepancies, or typing errors were followed up with specific centers for confirmation.

## Conclusion

This international survey offers a comprehensive view of current practices in OPT, reflecting both the field's maturation and its dynamic evolution since 2015. This equates to an average of 1800 patients per year. Consistent with the previous survey, most treatments continue to be carried out by a few well-established institutions with considerable experience in OPT and alternative treatments. Most of the centers adopted common protocols in dosimetry, total dose and fractionation, but significant variability persists in imaging, planning, QA, and delivery workflows.

While the ongoing adaptation of non-dedicated high-energy systems on gantries and integration into multipurpose treatment environments has broadened accessibility, some dosimetric parameters have been generally degraded, which requires a critical evaluation of clinical results with the new approaches. Maintaining a standardization for OPT, which encompasses beam quality, hardware, TPS, treatment delivery, and QA, is of paramount importance. The combination of a small target and adjacent sensitive critical OARs with unique immobilization considerations, coupled with submillimeter positioning and hypofractionation, is a challenge for curative radiation therapy. Due to the unique requirements for OPT, subtle differences in treatment quality may translate into significant differences in morbidity in ocular health and vision loss.

The obsolescence of proton systems and key planning tools and a lack of standardization remain pressing challenges, providing motivation for well-designed and economic commercial solutions suitable for a clinical environment. Nevertheless, growing interest in advanced imaging, automated workflows, and emerging modalities such as FLASH therapy and combined treatments with immunotherapy point to a promising future.

Continued collaboration, technological innovation, and clinical validation are essential to ensure the safe and effective delivery of OPT worldwide.

## Declaration of generative AI and AI-assisted technologies in the writing process

During the preparation of this work the authors used ChatGPT 5 to check grammar and reduce the length of some paragraphs. After using this tool/service, the authors reviewed and edited the content as needed and take full responsibility for the content of the published article.

## Declaration of Competing Interest

The authors declare the following financial interests/personal relationships which may be considered as potential competing interests: Jan-Willem Beenakker reports a relationship with RaySearch Laboratories AB that includes: funding grants. Jan-Willem Beenakker reports a relationship with Varian Medical Systems Inc that includes: funding grants. Jan-Willem Beenakker reports a relationship with Philips Healthcare Netherlands that includes: funding grants. Alejandro Mazal reports a relationship with Ion Beam Applications SA that includes: consulting or advisory, funding grants, speaking and lecture fees, and travel reimbursement. Helen Shih reports a relationship with National Association for Proton Therapy that includes: non-financial support. Helen Shih reports a relationship with Ion Beam Applications SA that includes: non-financial support. Helen Shih reports a relationship with PTCOG that includes: non-financial support. If there are other authors, they declare that they have no known competing financial interests or personal relationships that could have appeared to influence the work reported in this paper.

## Footnotes

1 Excludes two centers which reported FTE > 100, as this appeared to represent the whole proton team and not the team dedicated to ocular work
